# P338: Patient safety in Senegal: situation of the hospital regulations

**DOI:** 10.1186/2047-2994-2-S1-P338

**Published:** 2013-06-20

**Authors:** TS Ndiaye

**Affiliations:** 1 Faculty of Law and Political Sciences, Cheikh Anta Diop University, Dakar, Senegal

## Introduction

Care security issues are a major challenge for governments, health professionals, patients as well as for practitioners. The practice of medicine cannot ensure optimal security actors hospital in the absence of a legal framework defining the rules and procedures to be followed to prevent oversights and errors, provide care and a quality environment.

## Objectives

Conduct a situation analysis of the status of the regulations on the safety of patients and analyze the practical arrangements made on the ground at hospitals in Senegal.

## Methods

The methodology used is based on a series of surveys, interviews followed by a literature review at the hospital.

## Results

The results reveal a significant gap between the technical and regulatory. Despite the implementation of a national program to fight against NI and the existence of a central management of health facilities, very few codified rules have a direct impact on patient safety were identified.

## Conclusion

The legal aspects often poorly or not addressed expose virtually all hospitals in the litigation impacts disabling psychological and financial strengths. In the context of research quality and value for money of the rules governing hospital practices to ensure the safety of the patient must be taken. The establishment of rules codified by the authority, and protocols approved by all stakeholders contribute to better patient safety.

## Disclosure of interest

None declared

